# Supplementary material for: Prediction of risk of prolonged post-concussion symptoms: Derivation and validation of the TRICORDRR (Toronto Rehabilitation Institute Concussion Outcome Determination and Rehab Recommendations) score
Source: PLoS Med. 2021 Jul 8;18(7):e1003652. doi: 10.1371/journal.pmed.1003652 (PMC8266123; doi:10.1371/journal.pmed.1003652)
Supplement: S1 Table — (DOC) [file pmed.1003652.s002.doc]

| Codes for comordities of interest [Go Back](#APPENDIX_5_goback) | | | | |
| --- | --- | --- | --- | --- |
| **Comorbidity** | **Code Type** | **Code and Description** | | |
| Brain injury | OHIP | 850 Concussion  854 Other head injuries | | |
| Anxiety and mood disorders | OHIP | 296 Manic depressive psychosis, involutional melancholia  300 Anxiety neurosis, hysteria, neurasthenia, obsessive compulsive neurosis, reactive depression  301 Personality disorders (e.g., paranoid personality, schizoid personality, obsessive compulsive personality)  307 Habit spasms, tics, stuttering, tension headaches, anorexia nervosa, sleep disorders, enuresis  309 Adjustment reaction  311 Depressive or other non-psychotic disorders, not elsewhere classified | | |
|  | ICD-10 | F30 Manic episode  F31 Bipolar affective disorder  F32 Depressive episode  F33 Recurrent depressive disorder  F34 Persistent mood [affective] disorders  F38 Other mood [affective] disorders  F39 Unspecified mood [affective] disorder  F41 Other anxiety disorders  F42 Obsessive-compulsive disorder  F43 Reaction to severe stress, and adjustment disorders  F45 Somatoform disorders  F48 Other neurotic disorders  F60 Specific personality disorders  F99 Mental disorder, not otherwise specified | | |
| Headaches and migraines | OHIP | 346 Migraine  784 Headache | | |
| Psychosis-related disorders | OHIP | 295 Schizophrenia  297 Manic depressive psychosis, involutional melancholia | | |
|  | ICD-10 | F20 Schizophrenia  F21 Schizotypal disorder  F22 Persistent delusional disorders  F23 Acute and transient psychotic disorders  F24 Induced delusional disorder  F25 Schizoaffective disorders  F28 Other nonorganic psychotic disorders  F29 Unspecified nonorganic psychosis | | |
| Sleep disorders | OHIP | 307 Habit spasms, tics, stuttering, tension headaches, anorexia nervosa, sleep disorders, enuresis  729.1 Myositis plain and trauma  780 ……… Insomnia | | |
| Pain disorders | OHIP | 338 ……… Chronic Pain  729.1 Myositis plain and trauma  780 Convulsions, ataxia, vertigo, headache, except tension headache and migraine | | |
| TMJ disorders | OHIP | 524 Prognathism, micrognathism, macrognathism, retrognathism, malocclusion, temporomandibular joint disorders | | |
|  |  | | | |
| Vestibular disorders | OHIP | | 379 Other disorders of the eye  380 Otitis externa  381 Serous otitis media, eustachian tube disorders  382 Suppurative otitis media  383 Mastoiditis  384 Perforation of tympanic membrane  386 Meniere's disease, labyrinthitis  387 Otosclerosis  389 Deafness  780 Convulsions, ataxia, vertigo, headache, except tension headache and migraine  781 Leg cramps, leg pain, muscle pain, joint pain, arthralgia, joint swelling, masses |  |
| Neurological disorders | OHIP | | 331 Other cerebral degenerations  332……… Parkinson’s  333 ……… Abnormal movement disorders  340 ………Multiple Sclerosis  345 Epilepsy  349………Unspecified disorders of the nervous system  352 Disorders of other cranial nerves  377 Optic neuritis  434 ………Occlusion of cerebral arteries  805 Fractures and fracture-dislocations - vertebral column - without spinal cord damage  806 Fractures and fracture-dislocations - vertebral column - with spinal cord damage |  |
| Substance abuse | OHIP | | 303 Alcoholism  305 Tobacco abuse |  |

**Supplemental Table 1** Premorbid Health Conditions Diagnostic Codes.

Ontario Health Insurance Plan (OHIP)

International Classification of Disease (ICD)
