# Supplementary material for: Prediction of risk of prolonged post-concussion symptoms: Derivation and validation of the TRICORDRR (Toronto Rehabilitation Institute Concussion Outcome Determination and Rehab Recommendations) score
Source: PLoS Med. 2021 Jul 8;18(7):e1003652. doi: 10.1371/journal.pmed.1003652 (PMC8266123; doi:10.1371/journal.pmed.1003652)
Supplement: S2 Table — (DOC) [file pmed.1003652.s003.doc]

| Variable | | Odds Ratio | 95% Confidence Interval | p-value |
| --- | --- | --- | --- | --- |
| Sex (Female) | | 1.23 | 1.213 – 1.251 | <0.0001 |
| Age Group | 18-30 | 0.89 | 0.863 – 0.923 | <0.0001 |
|  | 31-40 | 1.20 | 1.140 – 1.269 | 0.0022 |
|  | 41-50 | 1.24 | 1.176 – 1.300 | <0.0001 |
|  | 51-60 | 1.03 | 1.104 – 1.220 | 0.114 |
|  | 61-80 | 1.15 | 1.113 – 1.180 | <0.0001 |
|  | >81 (Reference Group) | | | |
| Mental Health | Anxiety & Depression | 1.93 | 1.889 – 1.972 | <0.0001 |
|  | Personality Disorders | 1.79 | 1.647 – 1.936 | <0.0001 |
|  | Bipolar Disorder | 2.93 | 2.344 –3.670 | <0.0001 |
|  | Other | 1.56 | 1.515 – 1.611 | <0.0001 |
| Neurological Disorders | | 1.28 | 1.200 –1.357 | <0.0001 |
| Sleep Disorders | | 1.22 | 1.191 – 1.256 | <0.0001 |
| Pain Disorders | | 1.21 | 1.180 –1.249 | <0.0001 |
| Migraine | | 1.21 | 1.172 – 1.243 | <0.0001 |
| Headache | | 1.13 | 1.098 – 1.176 | <0.0001 |
| Vestibular Disorders | | 1.18 | 1.151 – 1.216 | <0.0001 |
| Temporomandibular Joint Dysfunction (TMJD) | | 1.24 | 1.080 – 1.423 | 0.0023 |
| Prior TBI | | 1.15 | 1.080 – 1.223 | <0.0001 |
| Location of Diagnosis (ED) | | 1.00 | 0.996 – 1.001 | NS |

**Supplemental Table 2** Univariate logistic regressions in the Prolonged Concussion Symptom Cohort (2008 – 2014)
