# Supplementary figures and images for: Prediction of risk of prolonged post-concussion symptoms: Derivation and validation of the TRICORDRR (Toronto Rehabilitation Institute Concussion Outcome Determination and Rehab Recommendations) score
Source: PLoS Med. 2021 Jul 8;18(7):e1003652. doi: 10.1371/journal.pmed.1003652 (PMC8266123; doi:10.1371/journal.pmed.1003652)

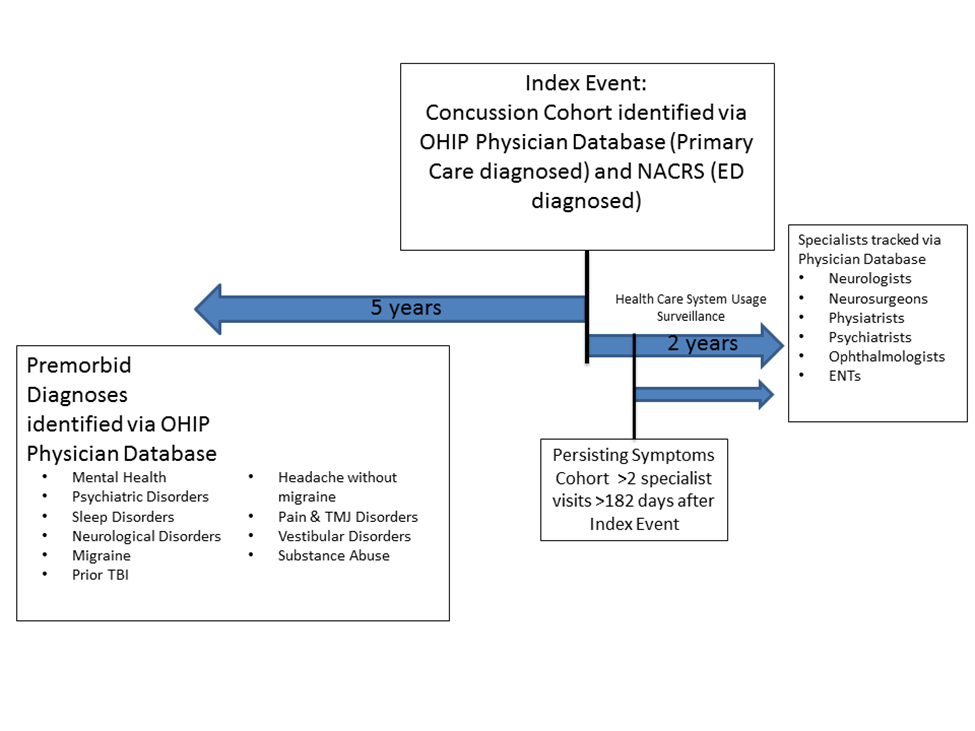

Supplement: S1 Fig — ED, emergency department; ENT, otolaryngology; NACRS; National Ambulatory Care Reporting System; OHIP, Ontario Health Insurance Plan; TBI, traumatic brain injury; TMJ, temporomandibular joint disorder. (TIF) [file pmed.1003652.s004.tif]

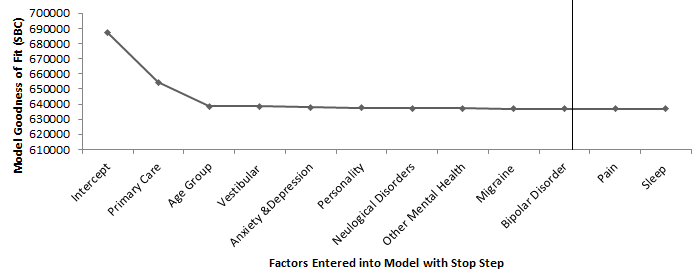

Supplement: S2 Fig — SBC, Schwarz Bayesian information Criterion for model goodness of fit; lower SBC better fit. (TIF) [file pmed.1003652.s005.tif]
