# Supplementary material for: Prediction of risk of prolonged post-concussion symptoms: Derivation and validation of the TRICORDRR (Toronto Rehabilitation Institute Concussion Outcome Determination and Rehab Recommendations) score
Source: PLoS Med. 2021 Jul 8;18(7):e1003652. doi: 10.1371/journal.pmed.1003652 (PMC8266123; doi:10.1371/journal.pmed.1003652)
Supplement: S1 Tool — (DOC) [file pmed.1003652.s006.doc]

**TRICORDRR (Toronto Rehabilitation Institute Concussion Outcome Risk Determination & Rehab Recommendations) Clinical Risk Tool**

|  | | SCORE |
| --- | --- | --- |
| AGE GROUP | 18 – 30 YEARS | -3 |
|  | 31 – 40 YEARS | -2 |
|  | 41 – 50 YEARS | -1 |
|  | 51 – 60 YEARS | 0 |
|  | > 61 YEARS | 4 |
| HOW OFTEN DID YOU SEE A PRIMARY CARE PHYSICIAN/ GP/ WALK-IN CLINIC IN PAST YEAR? | MORE OFTEN THAN ONCE A MONTH (>13 TIMES A YEAR) | 3 |
|  | ABOUT ONCE A MONTH (9 - 12 TIMES A YEAR) | 1 |
|  | ONCE EVERY FEW MONTHS (5 -8 TIMES A YEAR) | 0 |
|  | NEVER OR RARELY (0 – 4 TIMES A YEAR) | -1 |
| HAVE YOU BEEN DIAGNOSED WITH | ANXIETY and/or DEPRESSION | 1 |
|  | BIPOLAR DISORDER | 4 |
|  | OBESSIVE COMPULSIVE DISORDER (OCD) OR BEEN TOLD BY A PSYCHIATRIST YOU HAVE PERSONALITY ISSUES THAT MAY CAUSE PROBLEMS WITH YOUR PERSONAL LIFE AND/OR HEALTH | 3 |
| TOTAL SCORE | |  |

| Point | Risk | Point | Risk |
| --- | --- | --- | --- |
| -4 | 9% | 5 | 61% |
| -3 | 15% | 6 | 67% |
| -2 | 18% | 7 | 73% |
| -1 | 22% | 8 | 78% |
| 0 | 28% | 9 | 82% |
| 1 | 34% | 10 | 86% |
| 2 | 44% | 11 | 89% |
| 3 | 47% | > 12 | 91% |
| 4 | 54% |

**Supplemental Tool TRICORDRR (Toronto Rehabilitation Institute Concussion
Outcome Risk Determination and Rehab Recommendations)**
