# Supplementary material for: Prediction of risk of prolonged post-concussion symptoms: Derivation and validation of the TRICORDRR (Toronto Rehabilitation Institute Concussion Outcome Determination and Rehab Recommendations) score
Source: PLoS Med. 2021 Jul 8;18(7):e1003652. doi: 10.1371/journal.pmed.1003652 (PMC8266123; doi:10.1371/journal.pmed.1003652)
Supplement: S1 Text — (PDF) [file pmed.1003652.s007.pdf]

Data & Analytic Services

Dataset Creation Plan

General Description

**Project Name:**	Impact of Comorbid Conditions on Persistent Concussion Symptoms
**Description:**	Key Research Question:<br><br>How many people with an initial diagnosis of concussion or head injury from a family physician or paediatrician access neurological or rehabilitation specialized care? What are possible predictive factors for those that require further specialized care?<br><br>Research Objectives:<br><br>1) Analyze family physician and paediatrician OHIP billings to determine incidence of concussion by number with concussion as the diagnosis<br><br>2) Quantify the number of identified cohort that required further specialized care for post concussive symptoms by a specialist by following for 2 years after their diagnosis<br><br>3) Examine how co-morbid health conditions and previous health care seeking behaviours, as well as other demographic information could be predictive of those that require specialized care
**TRIM Number:**	2016 0970 031 000
**Client(s):**	Mark Bayley
**DAS Contact(s):**	Refik Saskin (refik.saskin@ices.on.ca or 416-480-4055 ext. 7470)<br>Nadia Gunraj (nadia.gunraj@ices.on.ca or 416-480-4055 ext. 2195)
**Assigned Analyst:**	Nadia Gunraj
**Budgeted Analyst Hours:**	
**Update History:**	2016-01-11 Reformatting and clarification (NG)<br>2015-11-12 Creation
**Type of study:**	Cohort

Cohort Definitions

Confirmed concussion:

Step	Description	# Excluded	Total Cohort
1	a) First ED visit with a diagnosis of concussion or head injury between 01JAN2008 and 31DEC2013 [APPENDIX 1] (NACRS)<br>OR<br>b) First visit to a GP/FP/paediatrician [spec IN ("00","26")] for concussion or head injury [dxcode IN: ("850","854")] between 01JAN2008 and 31DEC2013 (OHIP)<br>OR<br>c) First visit to a specialist [spec IN ("18","19", "23","24","31")] for concussion or head injury [dxcode IN: ("850","854")] between 01JAN2008 and 31DEC2013 and not identified in parts a) or b) (OHIP)<br>• Index date ≡ First date of visit<br>• Valid IKN<br>• For OHIP records<br>o Location = office / LTC / home		
2	No concussion or head injury [dxcode IN: ("850","854") or APPENDIX 1] in the year prior to index date (OHIP / NACRS)		
3	No acute care admission within 30 days after index date (DAD)		
4	Ontario residents (PCCF)		
5	Age 0-105 (RPDB)		
6	Available sex data (RPDB)		
7	Available LHIN data (RPDB)		
8	Death date missing or after index date (RPDB)		

... continued on next page

Cohort Definitions *(continued)*

Suspected concussion:

Step	*Description*	*# Excluded*	*Total Cohort*
1	First visit to GP/FP/paediatrician [spec IN ("00","26")] with trauma-related diagnosis [APPENDIX 2] between 01JAN2008 and 31DEC2013 (OHIP)<br>• Index date ≡ Date of first visit<br>• Valid IKN<br>• Location = office / LTC / home		
2	No visits for trauma-related diagnosis [APPENDIX 2] in the year prior to index date (OHIP)		
3	Patients not in confirmed concussion cohort (see definition above)		
4	At least one visit GP/FP/paediatrician (as defined in step 1) for neurological/cognitive complaints [APPENDIX 3] within the 6 months after index date (OHIP)		
5	No acute care admission within 30 days after index date (DAD)		
6	Ontario residents (PCCF)		
7	Age 0-105 (RPDB)		
8	Available sex data (RPDB)		
9	Available LHIN data (RPDB)		
10	Death date missing or after index date (RPDB)		

Variable Definitions

Baseline Characteristics:

- At index date:
  - Age (RPDB)
    - 0-4 years
    - 5-12 years
    - 13-17 years
    - 18-30 years
    - 31-65 years
    - 66-80 years
    - 80+ years
  - Sex (RPDB)
  - LHIN (PCCF)
  - Urban/rural indicator (PCCF)
  - Main specialty of physician [APPENDIX 4] (OHIP)
  - Season
    - Winter ≡ December to February
    - Spring ≡ March to May
    - Summer ≡ June to August
    - Fall ≡ September to November

Comorbidities:

- See APPENDIX 5
  - Number of comorbidities
    - 1-year lookback
    - 5-year lookback

… continued on next page

Variable Definitions

Additional Variables:

- Death date
- Continuum of care
  - GP only ≡ Patients diagnosed by GP/FP/ paediatrician only
  - ED only ≡ Patients diagnosed in ED only
  - GP=ED ≡ Patients visited both GP/FP/ paediatrician and ED
  - GP→ED ≡ Patients visited GP/FP/ paediatrician before visiting ED
  - ED→GP ≡ Patients visited ED before visiting GP/FP/ paediatrician

Outcome Definition(s)

Resource Use:

- In the 2 years following the index date
  - GP/FP/paediatrician visits
    - Total number of visits
  - Specialist visits [APPENDIX 4] (OHIP)
    - Total number of visits
    - First visit after index date
      - Wait time ≡ Time since index date
      - Main specialty of physician
      - Reason for visit (fee code) [APPENDIX 6]
    - Date of last visit
      - Main specialty of physician
      - Reason for visit (fee code) [APPENDIX 6]
  - Diagnostic imaging [APPENDIX 7] (OHIP)
    - Fee code
    - Number of services – one record per fee coded

Analysis Plan

Table 1. Baseline characteristics for the Confirmed and Suspected Concussion Cohorts

Characteristic	Confirmed Concussion			Suspected Concussion
	GP Only	ED Only	GP + ED	
	n=	n=	n=	n=
Index year, n (%)				
2008				
2009				
2010				
2011				
2012				
2013				
Age (years)				
Mean (± SD)				
Median (IQR)				
Age Group, n (%)				
<5 years				
5-12 years				
13-17 years				
18-30 years				
31-65 years				
66-80 years				
80+ years				
Sex, n (%)				
Female				
Male				
Patient LHIN, n (%)				
Erie St. Clair				
South West				
Waterloo Wellington				
Hamilton Niagara Haldimand Brant				
Central West				
Mississauga Halton				
Toronto Central				
Central				
Central East				
South East				
Champlain				
North Simcoe Muskoka				
North East				
North West				
Rural, n (%)				
Season, n (%)				
Winter				
Spring				
Summer				
Fall				
Main specialty of diagnosing physician, n (%)				
Family practice and general practice				
Neurology				
Psychiatry				
Ophthalmology				
Otolaryngology				
Paediatrics				
Physical medicine				

Analysis Plan *(continued)*

Table 2a. Baseline characteristics for the Confirmed Concussion Cohort, by LHIN

Characteristic	Overall N=	Erie St. Clair n=	South West n=	Waterloo Wellington n=	Hamilton Niagara Haldimand Brant n=	Central West n=	Mississauga Halton n=	Toronto Central n=	Central n=	Central East n=	South East n=	Champlain n=	North Simcoe Muskoka n=	North East n=	North West n=
Index year, n (%)															
2008															
2009															
2010															
2011															
2012															
2013															
Age (years)															
Mean (± SD)															
Median (IQR)															
Age Group, n (%)															
<5 years															
5-12 years															
13-17 years															
18-30 years															
31-65 years															
66-80 years															
80+ years															
Sex, n (%)															
Female															
Male															
Season, n (%)															
Winter															
Spring															
Summer															
Fall															

Analysis Plan *(continued)*

Table 2b. Baseline characteristics for the Suspected Concussion Cohort, by LHIN

Characteristic	Overall<br>N=	Erie St. Clair<br>n=	South West<br>n=	Waterloo Wellington<br>n=	Hamilton Niagara Haldimand Brant<br>n=	Central West<br>n=	Mississauga Halton<br>n=	Toronto Central<br>n=	Central<br>n=	Central East<br>n=	South East<br>n=	Champlain<br>n=	North Simcoe Muskoka<br>n=	North East<br>n=	North West<br>n=
Index year, n (%)															
2008															
2009															
2010															
2011															
2012															
2013															
Age (years)															
Mean (± SD)															
Median (IQR)															
Age Group, n (%)															
<5 years															
5-12 years															
13-17 years															
18-30 years															
31-65 years															
66-80 years															
80+ years															
Sex, n (%)															
Female															
Male															
Season, n (%)															
Winter															
Spring															
Summer															
Fall															

Analysis Plan

Table 3. Comorbidities occuring prior to the index date for the Confirmed and Suspected Concussion Cohorts

Comorbidity	Confirmed Cohort		Suspected Cohort	
	1-Year Lookback n=	5-Year Lookback n=	1-Year Lookback n=	5-Year Lookback n=
Brain injury, n (%)				
Anxiety and mood disorders, n (%)				
Headaches and migraines, n (%)				
Psychosis-related disorders, n (%)				
Sleep disorders, n (%)				
Pain disorders, n (%)				
TMJ disorders, n (%)				

Analysis Plan *(continued)*

Table 4a. Physician visits during the two years following the index date among patients in the Confirmed Concussion Cohort, by index year

Physician Specialty	Overall<br>N=	2008<br>n=	2009<br>n=	2010<br>n=	2011<br>n=	2012<br>n=	2013<br>n=
Number of GP/FP/paediatrician visits							
Mean (± SD)							
Median (IQR)							
Number of specialist visits							
Overall							
Mean (± SD)							
Median (IQR)							
General surgery							
Mean (± SD)							
Median (IQR)							
Neurosurgery							
Mean (± SD)							
Median (IQR)							
Plastic surgery							
Mean (± SD)							
Median (IQR)							
Neurology							
Mean (± SD)							
Median (IQR)							
Psychiatry							
Mean (± SD)							
Median (IQR)							
Ophthalmology							
Mean (± SD)							
Median (IQR)							
Otolaryngology							
Mean (± SD)							
Median (IQR)							
Paediatrics							
Mean (± SD)							
Median (IQR)							
Physical medicine							
Mean (± SD)							
Median (IQR)							
Optometrists							
Mean (± SD)							
Median (IQR)							

Analysis Plan *(continued)*

Table 4b. Physician visits during the two years following the index date among patients in the Suspected Concussion Cohort, by index year

Physician Specialty	Overall N=	2008 n=	2009 n=	2010 n=	2011 n=	2012 n=	2013 n=
Number of GP/FP/paediatrician visits							
Mean (± SD)							
Median (IQR)							
Number of specialist visits							
Overall							
Mean (± SD)							
Median (IQR)							
General surgery							
Mean (± SD)							
Median (IQR)							
Neurosurgery							
Mean (± SD)							
Median (IQR)							
Plastic surgery							
Mean (± SD)							
Median (IQR)							
Neurology							
Mean (± SD)							
Median (IQR)							
Psychiatry							
Mean (± SD)							
Median (IQR)							
Ophthalmology							
Mean (± SD)							
Median (IQR)							
Otolaryngology							
Mean (± SD)							
Median (IQR)							
Paediatrics							
Mean (± SD)							
Median (IQR)							
Physical medicine							
Mean (± SD)							
Median (IQR)							
Optometrists							
Mean (± SD)							
Median (IQR)							

Analysis Plan *(continued)*

Table 5a. Wait times for physician visits during the two years following the index date among patients in the Confirmed Concussion Cohort, by index year

Physician Specialty	Overall N=	2008 n=	2009 n=	2010 n=	2011 n=	2012 n=	2013 n=
Time to GP/FP/paediatrician visit (days)							
Mean (± SD)							
Median (IQR)							
Time to specialist visits (days)							
Overall							
Mean (± SD)							
Median (IQR)							
General surgery							
Mean (± SD)							
Median (IQR)							
Neurosurgery							
Mean (± SD)							
Median (IQR)							
Plastic surgery							
Mean (± SD)							
Median (IQR)							
Neurology							
Mean (± SD)							
Median (IQR)							
Psychiatry							
Mean (± SD)							
Median (IQR)							
Ophthalmology							
Mean (± SD)							
Median (IQR)							
Otolaryngology							
Mean (± SD)							
Median (IQR)							
Paediatrics							
Mean (± SD)							
Median (IQR)							
Physical medicine							
Mean (± SD)							
Median (IQR)							
Optometrists							
Mean (± SD)							
Median (IQR)							

Analysis Plan *(continued)*

Table 5b. Wait times for physician visits during the two years following the index date among patients in the Suspected Concussion Cohort, by index year

Physician Specialty	Overall<br>N=	2008<br>n=	2009<br>n=	2010<br>n=	2011<br>n=	2012<br>n=	2013<br>n=
Time to GP/FP/paediatrician visit (days)							
Mean (± SD)							
Median (IQR)							
Time to specialist visit (days)							
Overall							
Mean (± SD)							
Median (IQR)							
General surgery							
Mean (± SD)							
Median (IQR)							
Neurosurgery							
Mean (± SD)							
Median (IQR)							
Plastic surgery							
Mean (± SD)							
Median (IQR)							
Neurology							
Mean (± SD)							
Median (IQR)							
Psychiatry							
Mean (± SD)							
Median (IQR)							
Ophthalmology							
Mean (± SD)							
Median (IQR)							
Otolaryngology							
Mean (± SD)							
Median (IQR)							
Paediatrics							
Mean (± SD)							
Median (IQR)							
Physical medicine							
Mean (± SD)							
Median (IQR)							
Optometrists							
Mean (± SD)							
Median (IQR)							

Analysis Plan *(continued)*

To be discussed …

APPENDIX 1. ICD-10 diagnosis codes for concussion or head injury

Go Back

Code	Description
S00	Superficial injury of head
S01	Open wound of head
S02.0	Fracture of vault of skull
S02.1	Fracture of base of skull
S02.3	Fracture of orbital floor
S02.7	Multiple fractures involving skull and facial bones
S02.8	Fractures of other specified skull and facial bones
S02.9	Fracture of unspecified skull and facial bones
S04.0	Injury of optic nerve and pathways
S06	Intracranial injury
S07	Crushing injury of head
T02.0	Fractures involving head with neck
T90.2	Sequelae of fracture of skull and facial bones
T90.3	Sequelae of injury of cranial nerves
T90.4	Sequelae of injury of eye and orbit
T90.5	Sequelae of intracranial injury
T90.6	
T90.7	
T90.8	Sequelae of other specified injuries of head
T90.9	Sequelae of unspecified injury of head

APPENDIX 2. OHIP codes for trauma-related diagnoses Go Back

Code	Description
803	Fractures and fracture-dislocations - skull
805	Fractures and fracture-dislocations - vertebral column - without spinal cord damage
806	Fractures and fracture-dislocations - vertebral column - with spinal cord damage
807	Fractures and fracture-dislocations - ribs
808	Fractures and fracture-dislocations - pelvis
810	Fractures and fracture-dislocations - clavicle
812	Fractures and fracture-dislocations - humerus
813	Fractures and fracture-dislocations - radius and/or ulna
814	Fractures and fracture-dislocations - carpal bones
815	Fractures and fracture-dislocations - metacarpals
816	Fractures and fracture-dislocations - phalanges - foot or hand
821	Fractures and fracture-dislocations - femur
824	Fractures and fracture-dislocations - ankle
829	Fractures and fracture-dislocations - other fractures
831	Dislocations - shoulder
832	Dislocations - elbow
834	Dislocations - finger
839	Dislocations - other dislocations
840	Sprains, strains and other trauma - shoulder, upper arm
842	Sprains, strains and other trauma - wrist, hand, fingers
844	Sprains, strains and other trauma - knee, leg
845	Sprains, strains and other trauma - ankle, foot, toes
847	Sprains, strains and other trauma - neck, low back, coccyx
848	Sprains, strains and other trauma - other sprains and strains
869	Sprains, strains and other trauma - other head injuries
879	Sprains, strains and other trauma - lacerations, open wounds - except limbs
884	Sprains, strains and other trauma - lacerations, open wounds, traumatic amputations - upper limb(s)
894	Sprains, strains and other trauma - lacerations, open wounds, traumatic amputations - lower limb(s)
959	Sprains, strains and other trauma - other injuries or trauma

APPENDIX 3. OHIP diagnosis codes for neurological complaints Go Back

Code	Description
306	Neuroses and personality disorders - psychosomatic disturbances
307	Neuroses and personality disorders - habit spasms, tics, stuttering, tension headaches, anorexia nervosa, sleep disorders, enuresis
368	Eye - amblyopia, visual field defects
388	Ear and mastoid - wax or cerumen in ear, other disorders of ear and mastoid, tinnitus
780	Signs and symptoms not yet diagnosed - convulsions, ataxia, vertigo, headache, except tension headache and migraine
781	Signs and symptoms not yet diagnosed - leg cramps, leg pain, muscle pain, joint pain, arthralgia, joint swelling, masses

APPENDIX 4. Codes for specialists of interest Go Back

Code	Description
00	Family practice and general practice
04	Neurosurgery
08	Plastic surgery
18	Neurology
19	Psychiatry
23	Ophthalmology
24	Otolaryngology
26	Paediatrics
31	Physical medicine
56	Optometrists

APPENDIX 5 Codes for comordities of interest

Comorbidity	Code Type	Code and Description
Brain injury	OHIP	850............Concussion<br>854............Other head injuries
Anxiety and mood disorders	OHIP	296............Manic depressive psychosis, involutional melancholia<br>300............Anxiety neurosis, hysteria, neurasthenia, obsessive compulsive neurosis, reactive depression<br>301............Personality disorders (e.g., paranoid personality, schizoid personality, obsessive compulsive personality)<br>307............Habit spasms, tics, stuttering, tension headaches, anorexia nervosa, sleep disorders, enuresis<br>309............Adjustment reaction<br>311............Depressive or other non-psychotic disorders, not elsewhere classified
	ICD-10	F30............Manic episode<br>F31............Bipolar affective disorder<br>F32............Depressive episode<br>F33............Recurrent depressive disorder<br>F34............Persistent mood [affective] disorders<br>F38............Other mood [affective] disorders<br>F39............Unspecified mood [affective] disorder<br>F41............Other anxiety disorders<br>F42............Obsessive-compulsive disorder<br>F43............Reaction to severe stress, and adjustment disorders<br>F45............Somatoform disorders<br>F48............Other neurotic disorders<br>F60............Specific personality disorders<br>F99............Mental disorder, not otherwise specified
Headaches and migraines	OHIP	346............Migraine<br>784............Headache
Psychosis-related disorders	OHIP	295............Schizophrenia<br>297............Manic depressive psychosis, involutional melancholia
	ICD-10	F20............Schizophrenia<br>F21............Schizotypal disorder<br>F22............Persistent delusional disorders<br>F23............Acute and transient psychotic disorders<br>F24............Induced delusional disorder<br>F25............Schizoaffective disorders<br>F28............Other nonorganic psychotic disorders<br>F29............Unspecified nonorganic psychosis
Sleep disorders	OHIP	307............Habit spasms, tics, stuttering, tension headaches, anorexia nervosa, sleep disorders, enuresis<br>729.1..........Myositis plain and trauma<br>780 ……… Insomnia
Pain disorders	OHIP	338 ……… Chronic Pain<br>729.1..........Myositis plain and trauma<br>780............Convulsions, ataxia, vertigo, headache, except tension headache and migraine
TMJ disorders	OHIP	524............Prognathism, micrognathism, macrognathism, retrognathism, malocclusion, temporomandibular joint disorders

Vestibular disorders	OHIP	379............ Other disorders of the eye<br>380............ Otitis externa<br>381............ Serous otitis media, eustachian tube disorders<br>382............ Suppurative otitis media<br>383............ Mastoiditis<br>384............ Perforation of tympanic membrane<br>386............ Meniere's disease, labyrinthitis<br>387............ Otosclerosis<br>389............ Deafness<br>780............ Convulsions, ataxia, vertigo, headache, except tension headache and migraine<br>781............ Leg cramps, leg pain, muscle pain, joint pain, arthralgia, joint swelling, masses
Neurological disorders	OHIP	331............ Other cerebral degenerations<br>332……… Parkinson’s

		333 ……… Abnormal movement disorders<br>340 ………Multiple Sclerosis<br>345............ Epilepsy<br>349………Unspecified disorders of the nervous system<br>352............ Disorders of other cranial nerves<br>377............ Optic neuritis<br>434 ………Occlusion of cerebral arteries<br>805............ Fractures and fracture-dislocations - vertebral column - without spinal cord damage<br>806............ Fractures and fracture-dislocations - vertebral column - with spinal cord damage
Substance abuse	OHIP	303............ Alcoholism<br>305............ Tobacco abuse

APPENDIX 6. OHIP fee codes for follow-up physician visits Go Back

Code	Description
A100	General/Family physician emergency department assessment
A181	Neurology - complex medical specific re-assessment
A183	Neurology - medical specific assessment
A184	Neurology - medical specific re-assessment
A185	Neurology - consultation
A186	Neurology - repeated consultation
A188	Neurology - partial assessment
A190	Psychiatry - special psychiatric consultation
A195	Psychiatry - consultation
A230	Ophthalmology - orthoptic assessment
A231	Ophthalmology - neuro-ophthalmology consultation
A233	Ophthalmology - specific assessment
A236	Ophthalmology - repeat consultation
A243	Otolaryngology - specific assessment
A245	Otolaryngology - consultation
A246	Otolaryngology - repeat consultation
A252	Ophthalmology - initial vision rehabilitation assessment
A254	Ophthalmology - follow-up vision rehabilitation assessment
A313	Physical medicine & rehabilitation - medical specific assessment
A385	Neurology - limited consultation
A425	Physical medicine & rehabilitation - comprehensive physical medicine and rehabilitation consultation
A511	Complex physiatry assessment
A511	Physical medicine & rehabilitation - complex physiatry assessment
A680	Family practice & practice in general - initial assessment - substance abuse
A695	Psychiatry - neurodevelopmental consultation
A888	Emergency department equivalent - partial assessment
A895	Psychiatry - consultation in association with special visit to a hospital in-patient, long-term care in-patient or emergency department patient
A911	Special family and general practice consultation
A912	Comprehensive family and general practice consultation
A917	Sport medicine focused practice assessment
C005	Non-emergency hospital in-patient services - family practice & practice in general - consultation
C263	Non-emergency hospital in-patient services - paediatrics - medical specific assessment
C265	Non-emergency hospital in-patient services - paediatrics - consultation
G790	Acquired brain injury management - first day (neurosurgery)
H055	Emergency department - physician on duty - consultation
H313	Physical medicine & rehabilitation - rehabilitation counselling
K004	Family practice & practice in general - psychotherapy - 2 or more family members in attendance at the same time
K005	Family practice & practice in general - primary mental health care - individual care
K007	Family practice & practice in general - psychotherapy - individual care
K010	Family practice & practice in general - psychotherapy - additional units per member (maximum 6 units per patient per day)
K012	Family practice & practice in general - psychotherapy - 4 people

… continued on next page

APPENDIX 6. OHIP fee codes for follow-up physician visits *(continued)* Go Back

Code	Description
K013	Family practice & practice in general - counselling - first three units of k013 and k040 combined per patient per provider per 12 month period
K019	Family practice & practice in general - psychotherapy - 2 people
K020	Family practice & practice in general - psychotherapy - 3 people
K024	Family practice & practice in general - psychotherapy - 5 people
K025	Family practice & practice in general - psychotherapy - 6 to 12 people
K032	Specific neurocognitive assessment
K033	Family practice & practice in general - counselling - additional units per patient per provider per 12 month period
K037	Family practice & practice in general - fibromyalgia/chronic fatigue syndrome care
K040	Family practice & practice in general - counselling - where no group members have received more than 3 units of any counselling Paid under codes k013 and k040 combined per provider per 12 month Period
K041	Family practice & practice in general - counselling - additional units where any group member has received 3 or more units of any Counselling paid under codes k013 and k040 combined per provider per 12 Month period
K623	Family practice & practice in general - certification of mental illness - application for psychiatric assessment
K624	Family practice & practice in general - certification of mental illness - certification of involuntary admission
K629	Family practice & practice in general - certification of mental illness - all other re-certification(s) of involuntary admission including completion of appropriate forms

APPENDIX 7. OHIP fee codes for diagnostic imaging Go Back

Code	Description
G414	Routine EEG
G540	Prolonged EEG monitoring
X001	Diagnostic radiology - skull - four views
X009	Diagnostic radiology - skull - five or more views
X188	Diagnostic radiology - computed tomography - head - with and without IV contrast
X400	Diagnostic radiology - computed tomography - head - without IV contrast
X401	Diagnostic radiology - computed tomography - head - with IV contrast
X402	Diagnostic radiology - computed tomography - complex head - without IV contrast
X405	Diagnostic radiology - computed tomography - complex head - with IV contrast
X408	Diagnostic radiology - computed tomography - complex head - with and without IV contrast
