# Supplementary material for: Prediction of risk of prolonged post-concussion symptoms: Derivation and validation of the TRICORDRR (Toronto Rehabilitation Institute Concussion Outcome Determination and Rehab Recommendations) score
Source: PLoS Med. 2021 Jul 8;18(7):e1003652. doi: 10.1371/journal.pmed.1003652 (PMC8266123; doi:10.1371/journal.pmed.1003652)
Supplement: Attachment — Submitted filename: PLoS Med Response to Reviewers.docx [file pmed.1003652.s008.docx]

Dear Dr. Moyer,

We would like to thank the editors and the reviewers for their comments. We feel these revisions have further strengthened this manuscript. The responses are provided below and in a track changed copy of the manuscript. We hope we have addressed the reviewers’ comments to their satisfaction and look forward to hearing from the editor about publishing this manuscript in PLoS Medicine.

Sincerely,

Laura Langer

Requests from the editors:

1. If possible, please compare the definition for PCS against more conventional criteria in a subset of randomly selected patients (e.g. a few hundred) where the latter are available as an embedded validation of the approach, or at least to show how it performs in comparison to more conventional approaches.

There are several definitions of PPCS but no unifying or agreed upon criteria for symptoms or time since injury. The DSM-IV definition is symptoms persisting beyond 3 months and the table below outlines the most common diagnostic criteria. At least 1 visit 3 months after index date was 29.8% (22.1% if more than 1 visit) which is consistent with estimates from previous research that 75-80% of adults recover from concussion within 3 months, however only 73.3% of the cohort has had follow-up by a primary care physician by 3 months post-injury. WHO and Ontario Neurotrauma Foundation Guideline for Concussion/Mild Traumatic Brain Injury and Prolonged Symptoms in adults define persisting symptoms as at least 1 or more symptom 30 days following injury (in this cohort that identified 39.7% with at least 1 specialist visit 31 days or more after index date; 60.0% have had follow-up with primary care physician within 30 days post-injury). A disadvantage of using the 3-month time point in our study with administrative health data is that there is a possibility that the individual may not have had the chance to visit their primary care physician about their persisting symptoms and receive a referral for a specialist for follow-up care or specialist visit may be for pre-injury medical issues. The 6-month time point allows for a more conservative estimate as we think it more appropriate given the limitations of administrative health data.

|  | Criteria | % range meeting criteria from literature | % meeting criteria in the Cohort (specialist visit at >30 or >91 days respectively) |
| --- | --- | --- | --- |
| WHO (ICD-10) | Head trauma that results in loss of consciousness and at least 3/8 symptoms (headache, dizziness, irritability, sleep problems, memory problems, concentration problems, problems with stress or substance use, fatigue) within 30 days of injury | 30-40% | 39.7% |
| DSM-IV | Head trauma with neuropsychological impairments and symptoms present that were either not present or not as severe before injury (headache, fatigue, dizziness, sleep problems, anxiety and/or depression, changes in personality, or apathy) 3 months after injury | 10-30% | 29.8% |

2.Title: Please revise your title according to PLOS Medicine's style. Your title must be nondeclarative and not a question. It should begin with the main concept if possible. "Effect of" should be used only if causality can be inferred, i.e., for an RCT. Please place the study design ("A randomized controlled trial," "A retrospective study," "A modelling study," etc.) in the subtitle (ie, after a colon).

Title has been changed.

3.Data Availability statement: Unfortunately, the link ([https://urldefense.com/v3/__http://www.ices.on.ca/DAS__;!!CjcC7IQ!agIxV2Nh5WoYfJJSUov0Hi3J5owtCeE9_WHaf_Vl3vqmDpi75L_43nHMz6wzGABw$](https://urldefense.com/v3/__http:/www.ices.on.ca/DAS__;!!CjcC7IQ!agIxV2Nh5WoYfJJSUov0Hi3J5owtCeE9_WHaf_Vl3vqmDpi75L_43nHMz6wzGABw$) [ices[.]on[.]ca]) did not seem to work when tried. For the ICES dataset, please provide a specific DOI/accession number needed to identify the relevant dataset, and if possible provide a contact email address (note this cannot be a study author) to request access. For the dataset creation plan and analytic code, please either include these as supporting information files, or provide a link where they can be accessed (the contact point for these cannot be one of the authors).

The link was included in mandatory boilerplate statements but from 2019 and may no longer be active. As this dataset was part of the Data Analytics Services (DAS) branch of ICES, contact [das@ices.on.ca](mailto:das@ices.on.ca) and reference TRIM#201609760310000. The most up to date to my knowledge (I was only given 1 day to make local copies of my files before being sent to work from home in March) copy Dataset Creation Plan (DCP) has also been included now.

4.Abstract: Methods and Findings: Please start the first sentence of this section: “Data from a cohort study (Ontario Concussion Cohort study, 2008-2016; n=1,330,336) including all adults with a concussion diagnosis by either primary care physician… were used in a retrospective analysis” or similar.

Change made.

5.Abstract: Methods and Findings: Please include some background demographic information (e.g. population and setting, ages, etc).

Information included in abstract

6.Abstract: Methods and Findings: At line 43: “The total cohort was divided into derivation…” Please also note the derivation and validation cohorts were not randomly split but divided based on year of index event.

Clarification made.

7.Abstract: Methods and Findings: For the risk estimates for PPCS in the derivation cohort, please include the actual numbers underlying the percentages. At Line 49-50, please also describe the variables included in the models for the validation and derivation cohorts.

Variable list has been added and the absolute risk values are listed instead of the percentages.

8.Abstract: Methods and Findings: In the last sentence of the Abstract Methods and Findings section, please describe the main limitation(s) of the study's methodology.

Sentence added, line 52-54 (clean version)

9.Abstract: Conclusions: We suggest beginning the paragraph with “In this study, we observed that premorbid psychiatric conditions…” or similar.

Wording changed as requested

10.Author Summary: Why was this study done?: Please revise the third point to: “The goal of the study was to create a risk calculation for every adult…”

Change made

11.Author Summary: What did the researchers do and find?: Please change 587.057 to 587,057

Typo corrected

12.Author Summary: What do these findings mean?: We suggest combining the last two bullet points, and adjusting the language to avoid implying causality “Most adults with a concussion recover by 6 months following injury; however, our results suggest that pre-existing disorders are associated with increased likelihood of prolonged post-concussion symptoms.”

Changes made

13. Methods: Ethics section: Please mention whether the requirement for informed consent was waived by the ethics board that approved the study.

Waiver of consent statement included

14.Methods: Prospective analysis plan: Did your study have a prospective protocol or analysis plan? Please state this (either way) early in the Methods section.

Yes, there was a prospective analysis plan, this has now been included

a) If a prospective analysis plan (from your funding proposal, IRB or other ethics committee submission, study protocol, or other planning document written before analyzing the data) was used in designing the study, please include the relevant prospectively written document with your revised manuscript as a Supporting Information file to be published alongside your study, and cite it in the Methods section. A legend for this file should be included at the end of your manuscript.

The analysis plan is included with the DCP

b) If no such document exists, please make sure that the Methods section transparently describes when analyses were planned, and when/why any data-driven changes to analyses took place.

c) In either case, changes in the analysis-- including those made in response to peer review comments-- should be identified as such in the Methods section of the paper, with rationale.

15. Results: Please provide ORs, 95% CIs and p values for all relevant results presented in the main text. If adjusted analyses are presented, please also present the results from unadjusted models (these may be in a separate or supporting information table, and it should be noted in the text or figure legend when adjusted analyses are presented and which factors were included in the adjustment).

Results have been edited for accuracy, reviewed the regression and it was the unadjusted value that was included in the text. Models were not adjusted.

16. Discussion: Please revise the first sentence to begin: “To the best of our knowledge, this is the largest study to date…”

Wording changed

17. Discussion: Line 287: Please use square brackets for in text citations throughout.

Typo corrected

18. Discussion: Line 324: Please adjust the reference within the bracket to [7,8].

Typo corrected

19. Discussion: Line 349: Please clarify what is meant by the sentence: “This may be due to decreased neurocognitive reserve.”

Clarification included

20. Discussion: Line 374: Please clarify “effects of medico=legal issues”

Typo corrected, examples provided

21. References: Please use the "Vancouver" style for reference formatting, and see our website for other reference guidelines [https://urldefense.com/v3/__https://journals.plos.org/plosmedicine/s/submission-guidelines*loc-references__;Iw!!CjcC7IQ!agIxV2Nh5WoYfJJSUov0Hi3J5owtCeE9_WHaf_Vl3vqmDpi75L_43nHMz7CB4_j0$](https://urldefense.com/v3/__https:/journals.plos.org/plosmedicine/s/submission-guidelines*loc-references__;Iw!!CjcC7IQ!agIxV2Nh5WoYfJJSUov0Hi3J5owtCeE9_WHaf_Vl3vqmDpi75L_43nHMz7CB4_j0$) [journals[.]plos[.]org]

Corrected references

22. Table on Page 15: This appears to be the results of the Multivariate Logistic regression model in table format (from Figure 2). Please make this more clear (either incorporate into Figure 2 as a series of columns, or include a separate Table legend.

I was not able to get the table and forest plot together on the same page in portrait mode for a unified figure 2 at the time of submission and have now split them into a table (Table 2) and figure (#2).

23. Table 1 on page 32: Please use consistent terms “Concussion Cohort” vs. Adult Cohort vs. Adult Concussion Cohort. Please define the abbreviation for GP, TBI, and TMD. Please indicate values that are N (%) vs. value (SD), for example.

Corrections have been made

24. As mentioned by reviewer 2, please do present the demographics for the derivation vs. validation cohort, either in the main table or a supporting information file.

Further demographics have been included in Table 1a and 1b

24. Table 2: Please indicate “Primary Care” reflects numbers of visits.

Clarification has been made

25. Figure 2: Please provide an x axis label, and please provide a descriptive legend for the figure. Please define the abbreviation for TMJ.

Figure 2 has been updated

26. Supplemental Figure 2: Please note in the legend that the stop step is indicated by the vertical line. Please define “SBC” and include a general X axis label for the figure.

Clarifications have been made

27. Supporting information file TRICORDRR risk tool: Please spell out or define the abbreviation for OCD.OCD has been spelled out

28. Supporting information file: Thank you for including the full text of your article “Increasing Incidence of Concussion: True Epidemic or Better Recognition?” however, it is not necessary to include as it is cited in the reference list.

Noted

29. Checklist: Please ensure that the study is reported according to the Transparent reporting of a multivariable prediction model for individual prognosis or diagnosis (TRIPOD) or the most appropriate reporting guideline for your study, and include the completed TRIPOD checklist as Supporting Information. Please add the following statement, or similar, to the Methods: "This study is reported as per the Transparent reporting of a multivariable prediction model for individual prognosis or diagnosis (TRIPOD) statement (S1 Checklist)."

TRIPOD statement included in the Methods

The TRIPOD statement can be found here: [https://urldefense.com/v3/__https://www.equator-network.org/reporting-guidelines/tripod-statement/__;!!CjcC7IQ!agIxV2Nh5WoYfJJSUov0Hi3J5owtCeE9_WHaf_Vl3vqmDpi75L_43nHMz-xXGTIE$](https://urldefense.com/v3/__https:/www.equator-network.org/reporting-guidelines/tripod-statement/__;!!CjcC7IQ!agIxV2Nh5WoYfJJSUov0Hi3J5owtCeE9_WHaf_Vl3vqmDpi75L_43nHMz-xXGTIE$) [equator-network[.]org]

When completing the checklist, please use section and paragraph numbers, rather than page numbers.

Comments from the reviewers:

Reviewer #1: This is a very interesting and important paper that seeks to employ a large existing diagnostic health data system to determine factors associated with prolong post concussive symptoms.

The finings that behavioral health is associated with prolonged symptoms is not necessarily novel yet this paper expands this fining in a large population based setting. The limitations of the study are related to confirmation of diagnosis and unrecorded information as well as not using an a priori definition for post concussive symptoms.

1. It would be helpful for the authors to consider the fact that those with behavioral health co morbidities could be expressed as a series of somatic complaints and concerns. Might there be a population of people that had many of the ill defined PPCS symptoms preinjury or ascribed everything many of the concerns to the concussion that had existed previously

Thank you for the suggestion. The authors have considered that those with more somatic health behaviours may ascribe their symptoms to their concussion (the good old days bias) and not necessarily to pre-existing conditions. These patients would probably benefit from additional health care resources such as cognitive behavioural therapy or appropriate management of their pre-morbid conditions regardless, and this tool may flag they are at risk, particularly if they have higher health care usage before injury. This has been further elaborated upon in the Discussion.

2. The authors may wish to add additional co morbid that could be linked to health complaints- OSA and overall BMI for example

Thank you for this suggestion, however co-morbid information that would only be available at record level are not available to ICES (Ontario does not have electronic health records). Individual ICD codes are compressed into phenotypical groups and reported as such (the ICD codes used for pre-morbid conditions are available in the Supplemental Materials, Table 1)

3. The recent work of the TRACK TBI group may be a jumping off point for further discussion ie Nelson et al and Stein et al. ie the role of behavioral health and lack of resolution of symptoms in a prospectively collected cohort. In addition, Seabury et al speaks to the lack of ED follow up in a large group of those with brain injury. This paper creates an important point to consider is that the work in this paper may help to consider a " at risk cohort" that deserves clinical follow up early after injury

Thank you for these papers to consider. The Nelson et al study Recovery After Mild Traumatic Brain Injury in Patients Presenting to US Level I Trauma Centers: A Transforming Research and Clinical Knowledge in Traumatic Brain Injury (TRACK-TBI) Study included all people with mild TBI at level 1 trauma centers including some who were CT scan positive and they did characterize their population well at onset. They acknowledge this may not reflect the broader mild TBI population however they clearly identify that a significant proportion 53% were having difficulties at 12 months. This is indeed a higher number than ours and may reflect the fact that the individuals were severe enough for transfer to a trauma center. We excluded all people who were severe enough to be admitted to hospital for our data set as we considered them complicated mild TBI if they required admission. This suggests that our populations may not be exactly comparable.

The Stein study (Risk of Posttraumatic Stress Disorder and Major Depression in Civilian Patients After Mild Traumatic Brain Injury: A TRACK-TBI Study) also confirmed our findings that those with history of pre-existing mental health issues were at higher risk of developing complications from their Mild TBI of depression. This study also included 66% who were sever enough for admission to hospital and 24.5% who were admitted to ICU. Risk factors for probable depression at 6 months after mTBI included less education (adjusted odds ratio, 0.89; 95%CI, 0.82-0.97 per year), being black, self-reported psychiatric history and injury resulting from assault or other violence. Unfortunately, in our study we do not have race-based data or individual education data. The Stein group did not calculate a risk score although they clearly identified risk factors. The populations do appear to be different as well. The Track TBI Study team could also go further to complete a CART analysis to develop a risk score for prolonged symptoms.

In our study, unfortunately we were unable to access individual record level data and looked at administrative level data however we do note the frequency of primary care usage in the year proceeding injury could serve as a proxy for behavioural health. In this cohort as part of a health economics sub-study, the rate of follow-up care by primary care physicians of those identified in emergency department visits is 85%, however the median number of days between index date and primary care follow-up is 20 days. Interestingly the Seabury study: Assessment of Follow-up Care After Emergency Department Presentation for Mild Traumatic Brain Injury and Concussion: Results From the TRACK-TBI Study similarly found that only 52% had follow-up within 3 months so clearly follow-up is challenging and identifying those with unfavourable outcomes is key.

An aim of this tool is that is can be calculated quickly and easily, ideally at the time of diagnosis or soon thereafter. The sooner a patient’s risk score is calculated, the sooner those that would benefit from early intervention could receive the necessary referrals to specialists. Part of the Knowledge Translation plan once this paper is published is education about the tool to physicians including those in emergency departments.

3a. The authors may to also construct a low risk predictor given the above Seabury et al data

We agree that this is also important. Patients with a risk score of either -4 or -3 could be considered lowest risk. The 2 stratums are the lowest quartile of the population and consist of individuals under 40 years of age, no history of mental health issue, no psychiatric history, and have below average rates of primary care usage the year before their injury. This could serve as a low-risk predictor and these patients should be reassured of a good recovery outcome and that additional medical attention will most likely not be necessary. However, one clear limitation that we highlight is there may be those who are missed with our approach with the use of administrative health data, and it’s limitations in only being able to track relevant health care providers that do not submit billing to the provincial health authority (i.e., psychologists, physiotherapists, occupational therapists) and as a result of our 365 day wash-out for concussion diagnosis codes in primary care, there is a possibility that those that are considered good outcome may be misclassified if they were managed by other professionals.

Reviewer #2: "Predicting Prolonged Post-Concussion Symptoms using 1 the TRICORDRR (Toronto Rehabilitation Institute Concussion Outcome Determination and Rehab Recommendations)" describes the development and validation of a risk score to predict risk of needing prolonged concussion care. A cohort from the Ontario Concussion Cohort Study (2008-2016) was used, with the derivation cohort being from 2009-2013, and the validation cohort being from 2008 & 2014. Certain risk factors such as advanced age, bipolar disorder, high level of primary care visits per year, etc. were identified. An AUC of 0.64 was achieved for the model applied to the validation cohort, and AUC=0.79 for the derivation cohort with bootstrap resampling.

The results suggest moderate efficacy of the risk score model towards concussion care prediction. Additionally, some issues might be addressed:

1. In Lines 50 to 52, the sentence "The area under the curve (AUC) for the derivation model was 0.79, Validation model was 0.64, and the bootstrap was 0.79" seems slightly awkward. It appears that the same final model was validated in all cases; if so it might be phrased more similarly to "the AUC the final model was 0.79 on the derivation dataset... validation dataset...", etc. Moreover, from line 197, it seems that the derivation cohort and bootstrap are actually one and the same. This might be clarified.

Thank you for pointing out this is not clear. Lines 50-52 have been updated for clarity

2. In Line 182, the details of the univariate logistic regressions etc. involved in variable selection, as well as the details of the bootstrap resampling procedure, might be presented - possibly in supplementary material. This is especially if the derivation may not follow exactly from Sullivan et al. (2004).

Univariate details are available in the Supplemental Materials (Table 2). Variables included in the bootstrap and the number of random resamples were added to the Methods section.

3. For the CART tree presented in Figure 4, the predictions/outcomes at each node of the tree might also be presented (as referred to in Line 196, with reference to Sullivan et al., 2004), as appears to be standard practice.

We agree that the outcomes on the CART are typically presented, unfortunately in this case as some of the outcomes were in the 100,000s, SAS cut-off the final digits in some of the cases on the CART output and only displayed the first 4 to 5 digits of the outcome n. This dataset has tested, and in several cases exceeded, SAS’s processing capabilities especially when graphics were concerned.

This is part of the output from the CART in the Derivation Cohort and vetted by ICES.

4. In line 204, "duplicate records" might be defined in more detail. If it refers to individuals with multiple diagnoses of concussion (e.g. first concussion in 2008, then in 2013), was there a particular reason for exclusion instead of including the first occasion? If not, the treatment for such patients with multiple diagnoses might be described further, especially as they are then possibly represented in both the derivation and validation dataset.

Thank you for mentioning that this can better defined. “Duplicated records” as defined by ICES are those that contain records that contain identical variables indicating data enter error as well as visit counts as tracked by OHIP are limited to 1 visit per OHIP number for a diagnosis code for each physician in the province. This has been added to the methods. For those with multiple concussions diagnosis codes by a primary care physician OR any concussion diagnostic code by a primary care physician following an index diagnosis through the NACRS system in a period of 365 days following the first code being billed, this was done to ensure the database was not resampling the same person in order to calculate the incidence of concussion. After the 365-day period expired, the individual could sustain another concussion and be included in another year’s cohort. Previous concussion was one of the pre-morbid conditions included in the 5-year retrospective look back; it was not found to be a significant predictor of persisting symptoms when included with other covariates. Lines 144 and 152 now contain the updated information regarding duplicate records.

5. The choice of having the validation cohort being comprised of both diagnoses before (2008) and after (2014) the derivation cohort, might be explained further. While this seems that it would mitigate confounders (e.g. treatment conditions) across the study period to an extent, there remains a potential concern of validation on data prior to that used for derivation. As such, it might be considered to report the outcomes for 2014 separately from 2008 as well.

Thank you for this comment. 2008 and 2014 were purposefully selected for validation as they were expected to be the best and worst years and did not want it to influence the derivation cohort. Based upon the previous work on characterizing this cohort, 2008 concussions were lower in both public and healthcare professional awareness (this graph is from the supplemental from Langer et al 2020 JHTR) whereas 2014 was after the ONF guidelines for the treatment of concussion in adults were released and would be influencing treatment for concussion as well as greater public interest and more research being published about concussions. We agree that validating this in datasets which contain people with a concussion diagnosed prior to 2008 may not be valid. The results have been updated to include the specific percentages of those that met criteria for PPCS in both 2008 (12.3%) and 2014 (13.1%).

6. The demographics odds ratio table on Page 15 does not appear to be captioned.

This table was supposed to be part of figure 2 but the table and forest plot were not fitting together on the same page and are now separate table and figure with captioning. Sorry for any confusion.

7. For the corresponding demographics tables (Tables 1a & 1b), it might be considered to also present the figures separated according to derivation and validation cohorts, to determine as to whether they share similar distributions.

The table has been updated

8. The derivation of the risk point system (Line 260, Table 2) might be described in greater detail, possibly in the supplementary material. If possible, the performance difference between a (more complicated logistic) model estimate based on raw odds ratios, and the approximated risk point system, might be presented.

Thank you for these suggestions. More information has been provided on how the point system was derived. When the point calculation is compared to the equation using the raw coefficients to determine risk, there was high congruency between calculated scores, an example has been provided line 307. Unfortunately at this time, any additional complex models are not feasible. This dataset is housed on the same servers that are being used for the provincial COVID-19 modelling and as my project files are approximately 1.1 TB in size and models typically take 30 CPU hours to run (bootstrapping was almost 45 hours), I have been deprioritized.

9. In Line 268, Figure 6 is described as "the number of cases that had PPCS..."; might this be the proportion of cases instead?

This wording change has been made

10. The numerical details of the percentages/number of patients involved for each risk score strata in Figure 6 might be included in the supplementary material.

Thank you for this comment. The values were not included due to ICES privacy policy. Though the strata are reported in aggregate, 68 of the cells met ICES requirement for either partial or complete cell suppression and these data could not be exported

Reviewer #3: This manuscript reports the results of the study focused on analysis for predicting risk of prolonged postconcussion symptoms (PPCS). Specifically, the study uses the Toronto Rehabilitation Institute Concussion Outcome Determination and Rehab Recommendations (TRICORDRR) in their prediction model. Overall, the studies well done and likely to make an important contribution to the literature. The following comments are intended to help the authors improve the overall quality and impact of their manuscript.

1. The definition of PPC as used in this study, based on two or more specialist visits for concussion related symptoms more than six months after injury, is novel and unorthodox. Please provide justification for this definition.

We agree that the definition used for PPCS is novel. PPCS does not have a standard clinical definition nor a consensus within the literature or by practicing physicians. Our definition of 2 specialist visits would indicate a more persisting medical complaint especially if more than 1 specialist physician was consulted. This is particularly important when looking at the health care usage in people with mental health conditions before injury to ensure another specialist such as a physiatrist or ophthalmologist is seen in addition to a psychiatrist to strengthen the likelihood the patient is seeking care for the concussion and not pre-injury factors. Unfortunately in Ontario, there can be long wait times to access some specialists; the 6 month requirement was selected to both reduce the likelihood the visit was booked prior to the index date of the concussion and therefore unrelated to the concussion as well as allow for enough time for the patient to seek a referral for a persisting symptom and see the physician will allowing people sufficient time to be able to see primary care physician to obtain a referral. By 6 months after index date, 86.7% of the cases had had an appointment with a primary care physician. 50.3% of the cohort had a minimum of 1 visit to a specialist from the index date to 2 years afterwards (26.4% of them only had 1 visit) with a mean of 6.3 visits (11.6SD) and a median of 3 visits; 29.8% had a specialist visit 3 months or more after their index date (35.7% only had 1 visit).

2.	It would be helpful to understand how accurate the premorbid health retrospective review was for this study, relying on relevant ICD-9 diagnostic codes. Obviously, premorbid conditions emerged as important predictor variables in the model, so the reliability of the original data is critical. Please comment

Thank you for this comment. The ICD codes used to identify the pre-morbid conditions was included in the Supplemental Material in Table 1. The authors acknowledge that the accuracy of pre-morbid conditions is dependent upon physician and/or billing specialists filing out their billings correctly and as individual health records are not available to the authors, we cannot validate the 587,057 records. Psychiatric codes were also cross referenced in the Ontario Mental Health Reporting System where the diagnoses will be made by psychiatrists with extensive clinical experience with this population to increase accuracy.

3.	The greatest concern about this study has to do with the methodology used to calculate the rate of PPCS. That is, the calculation of the rate of prolonged postconcussion symptoms as 12.5% appears to be based on the denominator of only those patients that had two years of healthcare system tracking, not the original cohort of patients with concussion (n=1,330,336). My main concern is that this methodology artificially inflates the reported rate of PPCS. That is, many of the original concussed cohort patients may have had a spontaneous and complete recovery, which negated their need for any follow-up in the healthcare system after their concussion. If so, then the current estimated rate of 12.5% excludes them in his face only on the percentage of patients who pursue follow-up after their injury and continue to have prolonged symptoms at least six months after injury. The persisting cohort of 73,122 patients with persistent symptoms represents only 5.4% of the original concussed cohort of 1,330,336 patients. The different methodologies represent more than a twofold difference, 5.4% versus 12.5%. This issue is of major importance, as it has important bearing on the reported rate of PPCS from this study, which is likely to be cited frequently after publication. Please provide verification and justification for the methodology used.

Thank you for pointing out this is not clear. The total cohort contains both pediatric and adult cases as well as those diagnosed in 2015 and 2016, which due to delays in data reporting to ICES, 2 years of health care tracking were not available to the authors. The subcohort used for this risk score only contained adults diagnosed between 2008 and 2014, excluding 565,296 cases under 18 and 177,983 cases of adults diagnosed in 2015 and 2016, and is 44.1% of the total cohort. All cases between 2008 and 2014 had full 2 years tracking data available to the authors. The 12.5% we are reporting is on the lower end of estimates of PPCS, where it can range from 10% to 40%, and may be higher in actuality as concussion related healthcare visits to psychologists, physiotherapists, and other qualified health care providers that do not submit billing to OHIP could not be tracked using the administrative health databases nor did we track concussion treatment by primary care physicians (which include sports medicine physicians in Ontario) due to the dataset creation to examine incidence of concussion.

4.	With respect to the results of your production model, please comment on how these findings from your very large study are novel or highly consistent with the existing literature. Specifically, comment on novel findings from this study after the existing literature on age and premorbid psychiatric factors predicting risk of prolonged postconcussion symptoms after mild traumatic brain injury.

Probably the most novel findings are the increased usage of primary care increases risk of PPCS, most studies do not seem to collect this variable and it is not a part of common data elements, as well as the finding the elderly are at the greatest risk of PPCS. Most research excludes those over 80 and this is an under studied population; this is important finding with the context of the expected increase in the elderly in the next decade or so. Psychiatric and mental health conditions are consistently found to increase risk of PPCS and these findings are in line with the previous research just with an absolute risk value attached to just how much they influence developing PPCS.

5.	The modeling statistics, results, and graphic illustration of the findings are of good quality.

Thanks so much for this comment☺
