# Supplementary material for: Prediction of risk of prolonged post-concussion symptoms: Derivation and validation of the TRICORDRR (Toronto Rehabilitation Institute Concussion Outcome Determination and Rehab Recommendations) score
Source: PLoS Med. 2021 Jul 8;18(7):e1003652. doi: 10.1371/journal.pmed.1003652 (PMC8266123; doi:10.1371/journal.pmed.1003652)
Supplement: Attachment — Submitted filename: Reviewer Responses April 2021.docx [file pmed.1003652.s009.docx]

Dear Editorial Staff,

We have endeavoured to fully address the remaining reviewer comments.

1. *Title: Please revise your title according to PLOS Medicine's style. Your title must be nondeclarative and not a question. It should begin with main concept if possible. "Effect of" should be used only if causality can be inferred, i.e., for an RCT. Please place the study design ("A randomized controlled trial," "A retrospective study," "A modelling study," etc.) in the subtitle (ie, after a colon). We suggest Prediction of risk of prolonged post-concussion symptoms: Derivation and validation of the TRICORDRR (Toronto Rehabilitation Institute Concussion Outcome Determination and Rehab Recommendations) score” or something similar*.

Thank you for the suggestion, the title has been changed to Prediction of risk of prolonged post-concussion symptoms: Derivation and validation of the TRICORDRR (Toronto Rehabilitation Institute Concussion Outcome Determination and Rehab Recommendations) score.

2. *Data availability statement: Thank you for clarifying this, and please also include the reference number in the statement, alongside the ICES contact information (reference TRIM#201609760310000) if relevant for data requests*.

The TRIM information has been included

3. *Prospective analysis plan: Thank you for including the analysis plan as a supporting information file. Please reference the file in the Methods at Line 201 (S1_Analysis Plan). From looking at the analysis plan, it does not seem to describe in detail the specific analyses outlined in the study. In the Methods section, please fully describe when specific analyses were planned, and when/why any data-driven changes to analyses took place, including changes from peer review comments (these are described very generally at Lines 202-204*).

The analysis plan has been further elaborated upon in the methods. The Data Creation Plan that was previously was the most recent working copy and not the finalized one. As mentioned in the previous comments, we have had some limitations in access to all materials due to working remotely during the pandemic. We note that we did plan a multivariate analysis however derivation of the risk score was not in the initial analysis plan.

4. Abstract: Line 41: Should this be “retrospective analysis” rather than retroactive?

Thanks for the picking up this typo, the spelling has been changed to retrospective

5. *Abstract: Please double check grammar/sentence structure throughout- for example: “The total cohort was divided…” at line 44, and “Variables were selected a priori... “ at line 46.*

The abstract has been edited.

6. *Abstract: Methods and Findings: Please clarify the values given in parentheses at lines 50-52: “Highest risk estimates for PPCS derived in the Derivation Cohort were: >61 years (0.54), bipolar disorder (0.52), high level of primary care visits per year (0.46), personality disorders (0.45), and anxiety and depression (0.33).”*

*The abstract has been modified*

7. *Abstract: Line 54: Please define the abbreviation OHIP at first use.*

OHIP has been defined in the abstract as Ontario Hospital insurance plan

8. *Abstract: Conclusions: Please revise to avoid causal language: “In this study, we observed that premorbid psychiatric conditions were associated with increased risk of a prolonged recovery from concussion. Increased health care system usage and older age also were significantly associated with increased risk.”*

We have modified accordingly to “ In this study, we observed that premorbid psychiatric conditions, pre-injury health system usage and older age were associated with increased risk of a prolonged recovery from concussion.”

9. *Author summary: Please use sentence structure/punctuation for bullet points.*

The author summary has been edited.

10. *Author summary: We suggest revising under “What do these findings mean” to read: “The risk score may aid physicians treating adults with a concussion to quickly assess the patient’s absolute risk of a good outcome or a prolonged recovery and to be able to tailor the treatment plan as appropriate, such as encouraging return to aerobic exercise, education about concussion, timely referrals for specialized psychological care, etc.” if relevant, to reflect the conclusion ties back to the derivation of the risk score.*

Thanks for the suggestion, the wording has been changed

11. *Author summary: We suggest revising the final bullet point to avoid causal implications: “...psychiatric disorders and health care utilization are associated with increased risk of likelihood…”*

Wording has been revised to avoid causal implications

12. *Methods: Line 128-129: Can you please clarify this sentence: “Visible minorities accounted for 29.3% of the population.”*

The Statistics Canada definition of a Visible Minority has been included

13. *Results: Line 263-264: Please provide the p value for this, in addition to the 95% CIs, and please revise for causal language: The location of diagnosis (ED or primary care) was not significantly associated with the number or type of specialist visits for persisting symptoms (OR 1.00, CI 0.996 – 1.006).*

Thanks for pointing this out. The wording has been revised and the p-value has been included.

14. *Results: Line 310: Please clarify to “...hypothetical patient aged 50-60 years…”*

The wording has been revised.

15. *Results: Line 324: If it has not been previously spelled out in the text to this point, please fully define the abbreviation for TRICORDRR here at first use.*

Acronym has been spelled out.

16. *Discussion: We suggest opening with a discussion of the main findings of the study.*

The main findings of the study are now included in the opening paragraph.

17. *Discussion: Line 394: Please include a space between “aging” and the reference bracket. (Please also correct at line 402, and double check throughout).*

The spacing between brackets has been corrected

18. *Discussion: Line 419-420: Please revise to “...it has the highest number of visits with specialist physicians compared with other premorbid conditions…” Please also elaborate on the “decreased neurocognitive reserve” here.*

Wording has been changed and “decreased neurocognitive reserve” has been elaborated upon.

19. *Discussion: Line 434: Please capitalize Black in this sentence.*

Black has been capitalized

20. *Discussion: Future Directions: Please clarify that the TRICORDRR is “the tool” indicated, and also it would be helpful to include a sentence putting into context what these ongoing studies hope to inform/accomplish.*

This section has been updated.

21. References: Please double check all formatting. Please use the "Vancouver" style for reference formatting, and see our website for other reference guidelines [https://urldefense.com/v3/__https://journals.plos.org/plosmedicine/s/submission-guidelines*loc-references__;Iw!!CjcC7IQ!dOJjuwax3Gp8mPCqzlbgyxkIhJfWQMTm0N4dY2sFgw1GM2Y8nyp3oxr5RMIvsxGA$](https://urldefense.com/v3/__https:/journals.plos.org/plosmedicine/s/submission-guidelines*loc-references__;Iw!!CjcC7IQ!dOJjuwax3Gp8mPCqzlbgyxkIhJfWQMTm0N4dY2sFgw1GM2Y8nyp3oxr5RMIvsxGA$) [journals[.]plos[.]org] For example, please check journal name and punctuation in 11, 12, 13 (throughout).

References have been reviewed and corrected as required.

22. *Supplemental Figure 1: Please define all abbreviations used in the legend. If possible, please provide a short description in the legend to help illustrate the main point*.

The Figure has been updated

23. *TRIPOD checklist: Please include the locations in the text where each item can be found, rather than checking the points off the list. Please use section and paragraphs, rather than page numbers, to refer to locations. Please include a separate supporting information file Title/label for this (e.g. S1_Checklist).*

Line numbers are now included

24. *Figure 2: In the X axis label, please change to “Odds Ratio”*

Done.

25. *Figure 3: Please provide a descriptive legend for the figure, making it slightly more clear what point is illustrated*.

A legend has been included.

26. *Figure 4: Please provide a descriptive legend for this figure.*

A legend is included.

27. *Figure 5: Please provide a descriptive legend for the figure, making it slightly more clear what point is illustrated.*

A legend is included.

**Comments from Reviewers**:

Reviewer #2: We thank the authors for considering our previous comments. However, some concerns remain:

1. *Given that the motivation for selecting 2008 and 2014 for validation was specifically that "...they were expected to be the best and worst years and did not want it to influence the derivation cohort", it could be appropriate to (also) present their analyses separately, possibly in supplementary material. This is since neither were expected to represent typical performance, and it is uncertain as to whether their combination into a single validation set would represent the typical cohort either. It might be noted that this choice could have contributed to the reduced validation performance of the model, which might be considered as a limitation.*

Thanks for this comment, the Limitations section has been updated to be more clear on the limitation of the decision to use these 2 years may have had on the validation of the model.

Minor issues:

2. *The sentence beginning in Line 52 might be "The area under the curve (AUC) was 0.79 for the derivation model, 0.79 for bootstrap internal validation of the Derivation Cohort, and 0.64 for the Validation model*."

Sentence has been changed.

3. *For the CART figure, the explanation pertaining to SAS graphical limitations are acknowledged, but it would remain recommended to extract the figures corresponding to the nodes if at all possible (e.g. by writing the values of the corresponding variables to the output log)*

The CART output did not provide sufficient information to accurately deduce the node values from the 107 leaves in the tree and we would be estimating the values.

*Reviewer #3: I appreciate the authors' response to reviewer comments and their respective manuscript revisions. These modifications have significantly improved the overall quality and impact of the manuscript.*

Thank you so much for this comment.
